# Finding the optimal recall rate in breast cancer screening: results from the ROCS study

**DOI:** 10.1007/s00330-026-12370-5

**Published:** 2026-03-05

**Authors:** Daniëlle van der Waal, Craig K. Abbey, Eric Tetteroo, Tanya D. Geertse, Maartje J. A. Smid-Geirnaerdt, Ioannis Sechopoulos, Mireille J. M. Broeders

**Affiliations:** 1 Dutch Expert Centre for Screening (LRCB), Nijmegen, The Netherlands; 2 Department of Psychological and Brain Sciences, University of California-Santa Barbara, Santa Barbara, CA USA; 3 Department of Radiology, Amphia Hospital, Breda, The Netherlands; 4 Department of Medical Imaging, Radboud University Medical Center, Nijmegen, The Netherlands; 5 Technical Medicine Center, University of Twente, Enschede, The Netherlands; 6 IQ Health Science Department, Radboud University Medical Center, Nijmegen, The Netherlands

**Keywords:** Breast, Breast cancer, Mammography, Screening

## Abstract

**Objectives:**

In breast cancer screening, determining the optimal balance between the number of screen-detected cancer cases and false-positive recalls is essential. This study explored the relationship between these indicators for the Dutch Digital Mammography Screening Program and aimed to determine the optimal recall rate.

**Materials and methods:**

From March to June 2019, 21 screening radiologists provided continuous Probability-of-Malignancy (PoM) scores during their standard reading sessions. Scores ranged from ‘no suspicion’ (PoM = −100) to ‘highest suspicion’ (PoM = 100). Follow-up data were obtained in June 2024 and included recall decisions after double reading, outcomes of further assessments (false positive or screen-detected cancer), and interval cancer diagnoses. Recall–detection and receiver operating characteristic (ROC) curves were constructed for each reader and averaged to obtain summary curves, with error bars computed from hierarchical bootstrapping of cases within readers (1000 resamples). The overall screening performance was quantified using the area under the ROC curve (AUC).

**Results:**

The final dataset comprised 40,829 screening records with 315 cancer cases. The overall recall rate was 2.33%, and the cancer detection rate after double reading was 6.4 per 1000 screens. Radiologist performance was high (AUC = 0.91). Moving the operating point results in either substantially lower cancer detection or relatively little gain.

**Conclusion:**

This prospective study identified the trade-off between unconditional recall and detection rates, as well as conditional sensitivity and specificity. We found that Dutch screening radiologists perform at a high level and operate at a point that seems to optimize the false-positive recall and cancer detection rate trade-off.

**Key Points:**

***Question*** *Breast cancer screening requires a good balance between detection and false-positive rate. However, the interrelationship between these rates, and thus the optimal recall, is unknown.*

***Findings*** *Overall, the Dutch screening radiologists performed with high accuracy, and the current operating point optimizes the trade-off between cancer detection and false-positive recalls*.

***Clinical relevance*** *ROCS provides an efficient method to evaluate performance and determine target values based on data from screening practice. This method applies to various screening programs. Screening evaluation is essential for achieving and maintaining a positive benefit-to-harm ratio for participants*.

**Graphical Abstract:**

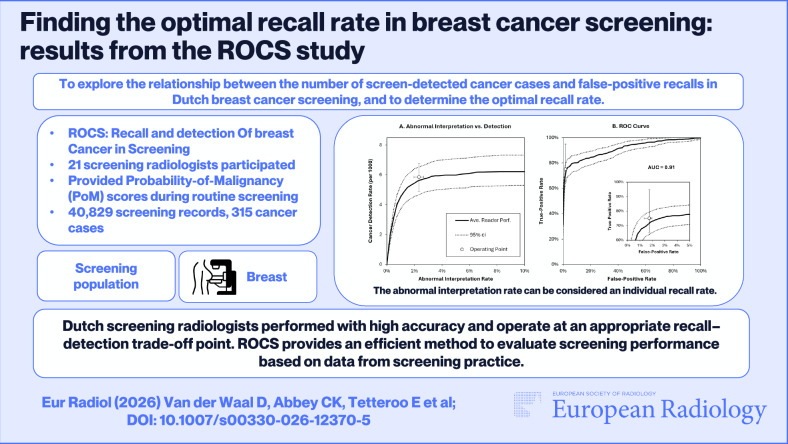

## Introduction

Mammography screening is known to decrease breast cancer mortality [1]. This, however, comes at a price, as screening harms include false-positive recalls [2]. To maximize the net screening benefit, it is important to find the optimal balance between the number of screen-detected cancers and false-positive recalls.

Recalling more screening participants should result in a higher cancer detection rate if the correct participants are recalled. This can be achieved by lowering the recall threshold, that is, recalling cases with less suspicious mammographic characteristics. However, this inevitably leads to more false-positive recalls as well. False-positive recalls are associated with increased anxiety and higher healthcare costs, and may disincentivize women from participating in subsequent screening rounds [3–5].

The number of false-positive recalls considered acceptable to find one additional cancer varies, which is reflected by the wide range of recall rates on an international level [6]. Recall rates are traditionally very low in the Netherlands [7]. In 2005, a simulation study by Otten and colleagues assessed the balance between cancer detection and false positives in Dutch screening, with the aim of optimizing recall [8]. The Otten study showed that, at the time, a significant number of screen-detected cancers could be gained by increasing recall, while the increase in false-positive recalls would be limited. However, if the recall rate increased too much, the benefit would level off, and the balance would become less favorable. Based on this study, Dutch screening radiologists were advised to increase the recall from 1% to approximately 2%.

Since this previous study, changes have been introduced in breast cancer screening, which may affect the balance between false positives and cancer detection. An example of a transition causing relevant changes is the one from film-screen to digital mammography [9, 10]. In addition, the study by Otten and colleagues has several limitations [8]. The retrospective design and laboratory setting raise the question of the extent to which the results are applicable to real-life screening practice. Therefore, a new prospective study, the Recall and detection Of breast Cancer in Screening (ROCS) study [11], started in 2019. This study was based on full-field digital mammography, unlike film-screen mammography in the study by Otten and colleagues [8]. ROCS explored the relationship between breast cancer detection and false-positive rates for the Dutch Breast Cancer Screening Program and aimed to determine the optimal recall rate. The data acquisition method developed for ROCS allows for a minimally invasive and inexpensive way to assess screening performance in practice that can be applied to other screening programs.

## Materials and methods

### Study design

This was a prospective cohort study, in which data were collected within the Dutch Breast Cancer Screening Program. In this Program, screening participants automatically consent to the use of their data for research purposes, unless they opt out. Ethical approval was waived by the local ethics committee of the Radboud University Medical Center (CMO Radboudumc; file number: 2018-4991, confirmation received on February 13, 2019). The STrengthening the Reporting of OBservational studies in Epidemiology (known as STROBE) statement was followed in reporting this study.

### Setting

In the Netherlands, women between the ages of 50 and 75 years are invited to participate in full-field digital mammography screening, which is executed by a single national screening organization. The intended screening interval is two years. All screening examinations are double-read. In the event of a discrepancy, the second reader rereads the screening exam with knowledge of the first reader’s decision, the first and second readers attempt to reach consensus, or the exam is sent to an independent third reader (arbitration).

### Data collection

From March to June 2019, 21 screening radiologists (5 out of the 15 national reading units) participated in ROCS. They performed normal individual screening assessments during standard reading sessions. In addition, in the context of this study, they also gave each examination a Probability-of-Malignancy (PoM) score. This has previously been described in detail [11]. Briefly, radiologists were shown a continuous scale on a tablet, ranging from ‘no suspicion’ (green area on the left side of the scale, PoM = −100) to ‘highest suspicion’ (red area on the right side of the scale, PoM = 100). This scale was based on previous work [12]. The PoM score does not directly indicate the likelihood of a cancer diagnosis but can instead be used to rank exams from low to high suspicion of malignancy. The radiologists could provide a PoM score by swiping over the desired point on the scale. When the radiologists swiped, the tablet automatically took a picture of the pseudonymized ID that appeared on the reading workstation, which could be linked to the screening participant by the screening organization.

The radiologists were instructed to use the full scale and ensure that a recall recommendation resulted in a PoM score higher than 0. In addition, radiologists were asked to score only the screening examinations where they acted as the first or second reader (i.e., no arbitration readings). Each screening examination was scored as a whole, resulting in one PoM score per screening examination per radiologist. If radiologists wanted to revise their score, for example, after making a mistake, they could swipe again, and only the last score given per screening exam per radiologist would be used in the analyses for these sequential duplicates. Furthermore, if both the first and second readers of a screening examination participated in our study, both PoM scores were included in our final database. Therefore, there was ultimately a maximum of one PoM score per radiologist for each screening examination in our analyses, and a maximum of two PoM scores per screening examination from different radiologists.

After the baseline data collection, the pseudonymized IDs visible in the pictures were entered manually and double-checked. Subsequently, these numbers were verified using data from the screening organization, and typographical errors were corrected. In June 2024, the screening organization provided follow-up data related to these examinations. The follow-up data consisted of the individual recall recommendations of the radiologists, whether the participants were recalled after double reading, conclusions after further diagnostic assessments (false-positive recall or screen-detected cancer), and interval cancer diagnoses. In a small number of cases, the screening organization was unable to share follow-up data. This happened when there was a technical problem with the case ID photographed by the ROCS system camera, when the radiologist had acted as a third reader in a screening examination, or if the participant had selected to opt out of sharing data. At no point did the screening organization have access to readers’ PoM scores.

During data cleaning, PoM scores were removed for which the screening organization could not provide follow-up data or for which follow-up data were incomplete. In addition, for sequential duplicate scores (i.e., revised PoM scores given by the same screening radiologist for the same screening exam), the first score was removed. For nonsequential duplicate scores, the last score was removed, as this could potentially be a consensus reading. Finally, two reading sessions that appeared to largely consist of duplicate scores were removed.

### Statistical methods

We use the terms “abnormal interpretation rates” and “abnormal detection rates” to refer to single-reader results, which are the focus of this study. The abnormal interpretation rates in the performance curves are based on the PoM scores, using different cutoff points to label a screening exam as abnormal. This can be interpreted as an individual recall rate. When determining abnormal detection rates, all cancers, both screen-detected and interval, that were diagnosed within two years of abnormal interpretation were included. The standard terms “recall rate” and “false positive recall” characterize the final screening outcomes, involving the two-reader interpretations and any consensus or arbitration steps.

The prespecified primary endpoint of the study is an estimate of the average trade-off curve between the abnormal interpretation rate and cancer detection rate for single-reader screening interpretations. The targeted sample sizes of 20 readers and 2000 cases per reader were determined by a pre-study power calculation [11]. A number of additional secondary endpoints are also reported here: the observed operating point from the screening organization data, the receiver operating characteristic (ROC) curve comparing false-positive rate to sensitivity and its area under the curve (AUC), and estimates of the change in abnormal cancer detection rate as a function of a change in abnormal interpretation rate.

Hierarchical bootstrapping was used to model uncertainty arising from the random sampling of readers and cases in the study, reported as 95% confidence intervals on screening performance estimates and 95% pointwise confidence bands for estimated curves. The bootstrapping procedure involves an initial resampling of readers, followed by independent resampling of the cases nested within each reader. Note that the comparison of abnormal cancer detection rate differences as a function of abnormal interpretation rate differences is performed on a within-reader basis, and thus, no reader resampling was performed for this curve.

## Results

**Fig. 1:**
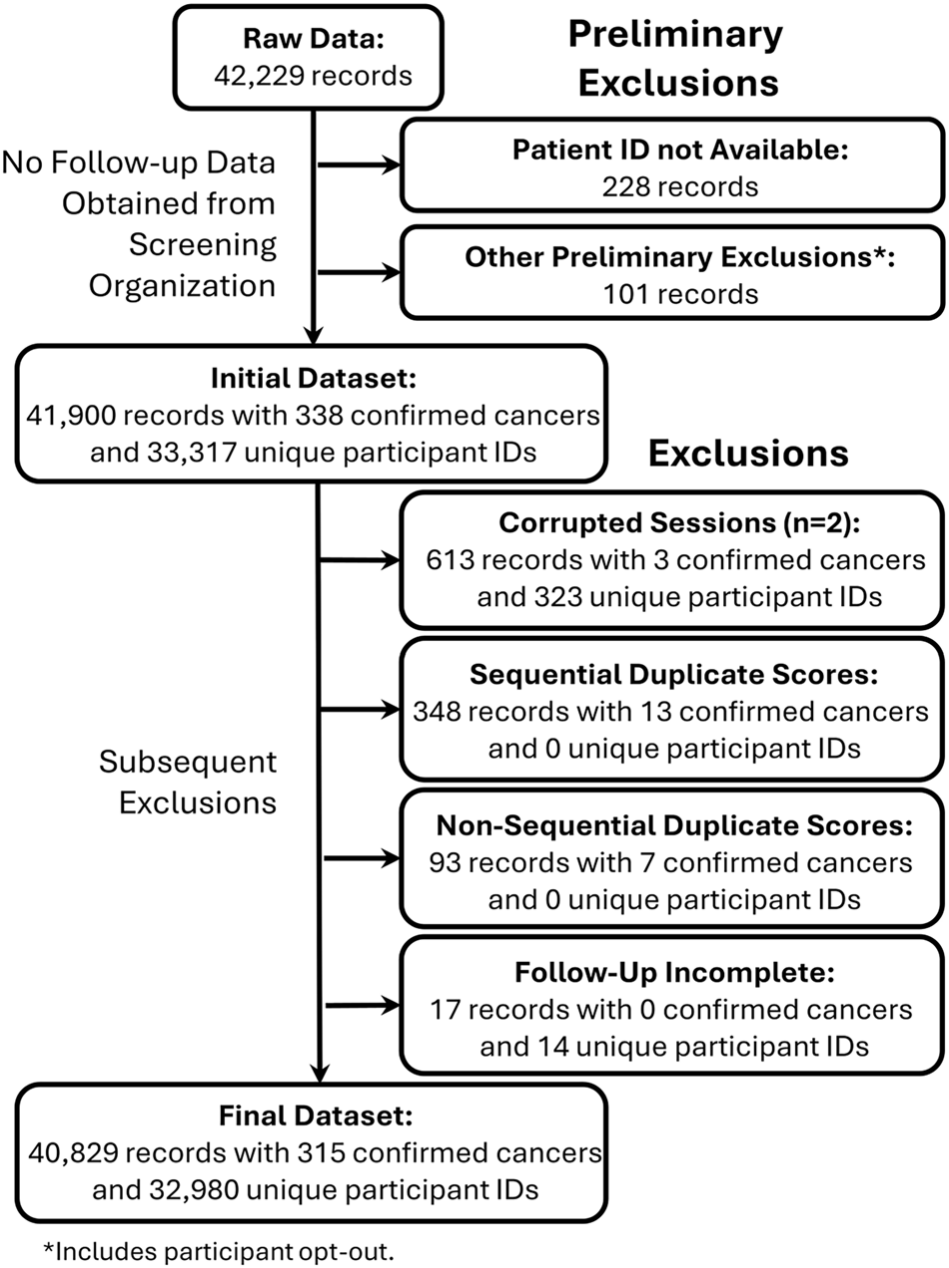
Exclusions applied to raw ROCS data. The preliminary exclusions were records for which no follow-up data could be provided by the screening organization. For each step in the subsequent exclusions, the number of eliminated records, confirmed cancers, and unique participant IDs from the final dataset are given

The initial data consisted of 42,229 PoM scores and individual screening assessment records from 21 readers in 198 reading sessions (Fig. 1). The number of included reading sessions per radiologist varied from 5 to 14, with a median of 10. The initial exclusions, for which no follow-up data were available, removed 329 records from the dataset, yielding a total of 41,900 records remaining. Subsequent data cleaning excluded 1071 records for a final total of 40,829 records based on examinations of 32,980 screening participants. Thus, 7849 screening exams appeared twice in the final dataset, as both the first and second readers assessed the same exam and provided a PoM score as part of this study. In total, there were 315 cancer diagnosis records, originating from 256 participants who were diagnosed with breast cancer within two years after the baseline screening examination. Table 1A summarizes the size and cancer rates in the ROCS data.

**Table 1:** Overview of the ROCS data

Quantity	*N*	Rate
A. ROCS data size		
Total records	40,829	-
Unique pseudonymized IDs	32,980	-
Records with cancer	315	7.7 per 1000 records
Pseudonymized IDs with cancer	256	7.8 per 1000 IDs
Pseudonymized IDs appearing twice	7849	23.8% of the IDs
Radiologists providing scores	21	-
B. Reading performance		
Abnormal interpretations^a^	921	2.26% of the records
Abnormal detections^a^	236	5.8 per 1000 records
Recall cases^b^	769	2.33% of the IDs
Recall detections^b^	210	6.4 per 1000 IDs

A: summary of the ROCS data size. B: overview of the reading performance in ROCS

^a^ Abnormal interpretations and detections are all records, and the subset of records with verified cancer, identified as abnormal at single-reader interpretation

^b^ Recall cases and detections are all cases and verified cancer cases identified for recall at consensus

### Overall reading performance based on screening data

As shown in Table 1B, 921 of the 40,829 individual screening assessments in the ROCS dataset involved an abnormal interpretation (i.e., the individual screening radiologist gave a recall recommendation). This resulted in an abnormal interpretation rate of 2.26%. Among these abnormal interpretations, 236 records involved breast cancer, resulting in an abnormal detection rate of 5.8 per 1000 interpretations. It should be noted that these were not all screen-detected cancers. There were two abnormal interpretations that did not result in a recall decision after consensus but were subsequently diagnosed in the 2-year screening interval.

After double reading, 769 of the 32,980 screening participants were ultimately recalled, resulting in an overall recall rate of 2.33%. There were 210 screen-detected cancers, which equated to a detection rate of 6.4 per 1000.

### Distribution PoM scores

**Fig. 2:**
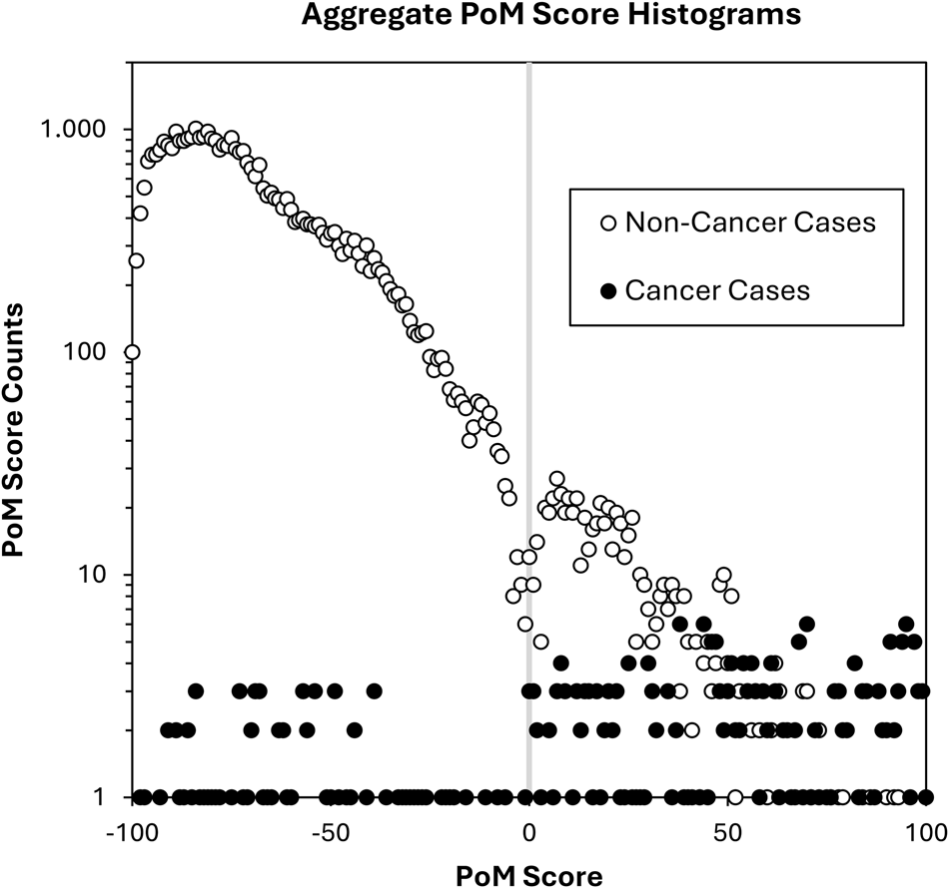
PoM score histograms. Aggregate responses for cancer and non-cancer cases are plotted over the range of possible PoM values (−100 to 100). The midpoint (gray line) indicates the threshold for an abnormal interpretation. Scores with 0 counts are not plotted. The dip in negative responses suggests that there is some avoidance near this value. Non-cancer case responses are heavily skewed towards negative PoM scores, and cancer cases are moderately skewed towards positive PoM scores

Figure 2 shows the frequency of all PoM scores (*n* = 40,829) over the entire range of possible scores (−100 to 100), subdivided into cancer and non-cancer cases. Both non-cancer and cancer cases were assigned scores across the entire range. However, non-cancer cases are heavily skewed towards negative scores, and cancer cases are moderately skewed towards positive scores.

### Performance curves

**Fig. 3:**
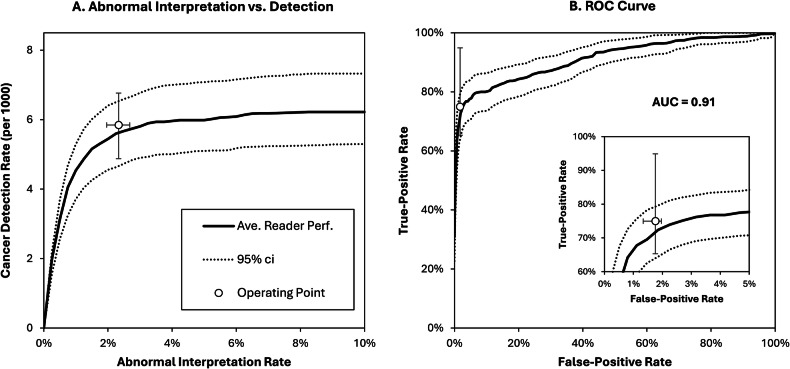
Performance curves. Plots of the unconditional (**A** abnormal interpretation and cancer detection rates) and conditional (**B** true-positive and false-positive rates) performance curves. Legend applies to both plots. The operating point is defined from the screening data. Confidence bands and intervals represent 95% intervals derived from 1000 bootstrap replications of the cases. The insert in the ROC curve shows a magnified view of the curve near the operating point. Across both plots, there is a rapid drop in cancer detection and true-positive rates for abnormal interpretation and false-positive rates below the operating point. Please note that the axes in the inset figure have a different scale

Figure 3A shows the cancer detection rate plotted against the abnormal interpretation rate. Initially, at an abnormal interpretation rate between 0% and 2%, there is a strong increase in cancer detection as the abnormal interpretation rate increases. At an abnormal interpretation rate between 2% and 3%, the increase in cancer detection levels off. This is also where the average radiologist’s operating point is currently located, with an abnormal interpretation rate of 2.26%. Supplementary Fig. 1 shows the curvature of the abnormal interpretation–detection graph (Fig. 3A), which further demonstrates that the radiologists’ operating point is located right at the end of the high negative curvature region.

The ROC curve (Fig. 3B) shows that the radiologists were able to distinguish cancer cases from non-cancer cases, with an AUC of 0.91. Similar to the previous graph, the current operating point is located at a point of strong curvature.

**Fig. 4:**
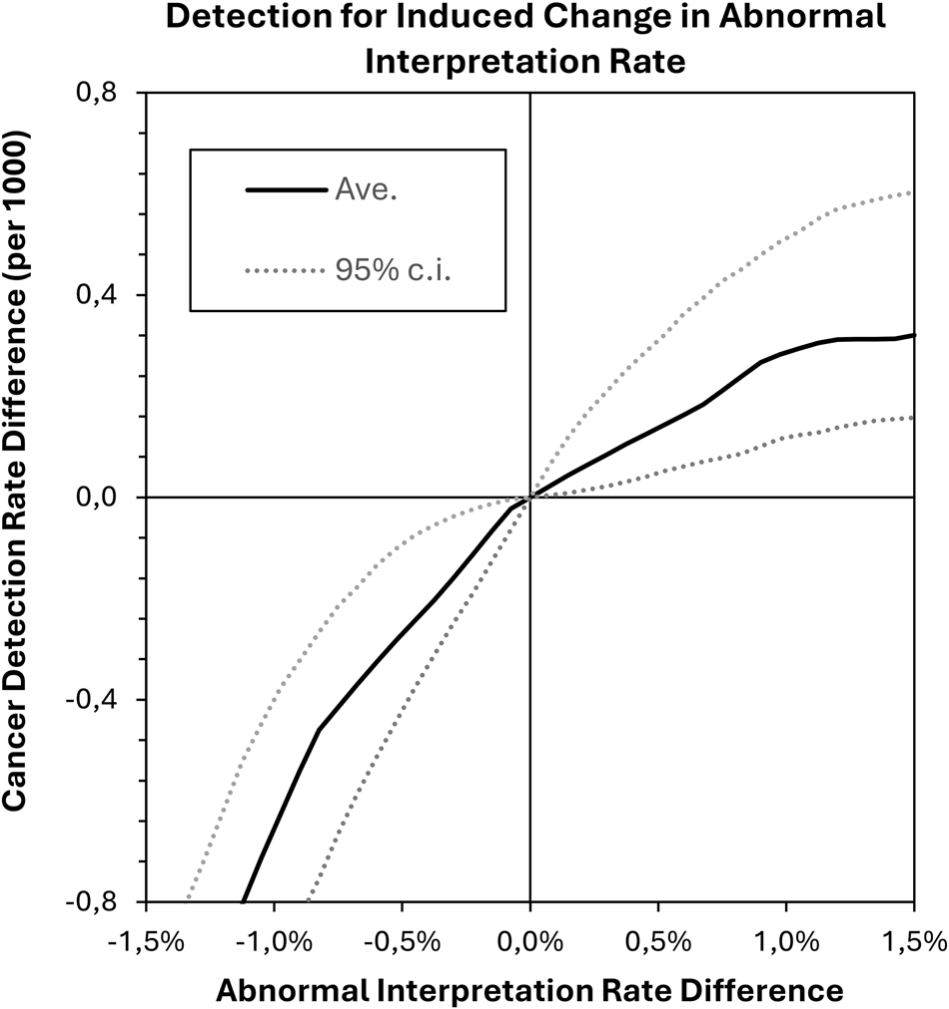
Performance trade-offs. The plot shows the predicted effect of an induced change in the abnormal interpretation rate. The 95% confidence band is derived from 1000 bootstrap replications across cases

The predicted change in the cancer detection rate due to an induced change in the abnormal interpretation rate is shown in Fig. 4. The zero point represents the current average abnormal interpretation and cancer detection rates. An increase of 0.5% in the abnormal interpretation rate is predicted to result in an increase in cancer detection of 0.14 per 1000 screening examinations. In contrast, a decrease in the abnormal interpretation rate of 0.5% compared to the operating point would lead to a more substantial decrease in cancer detection of 0.27 per 1000 screening examinations. Furthermore, an increase in the abnormal interpretation rate of 1% yields an additional cancer detection of 0.29 per 1000 (compared to baseline), while a 1% decrease results in a decrease in cancer detection of 0.65 per 1000.

## Discussion

This is the first large prospective study using data from real-life screening practice to evaluate the balance between cancer detection and false positives within the Dutch Breast Cancer Screening Program. This study showed that the screening radiologists performed well overall, with an AUC of 0.91. The results further show that Dutch screening radiologists, with an abnormal interpretation rate of 2.26%, currently operate at just above a region of high-curvature point when plotting the cancer detection rate against the abnormal interpretation rate (see Supplementary Fig. 1). This suggests that reducing the abnormal interpretation rate, and thus the number of recall recommendations, could substantially lower cancer detection. For example, if the abnormal interpretation rate decreases by 1%, the detection rate decreases by 0.65 per 1000. In contrast, increasing the abnormal interpretation rate by up to 1% may improve cancer detection, but by a smaller fraction (0.29 per 1000).

The curve plotting cancer detection against abnormal interpretation rate in the current study is similar to that found in the previous Otten study investigating Dutch screening performance [8]. Remarkably, in both studies, the curve levels off at a recall rate, or abnormal interpretation rate, between 2% and 3%. This is despite more than 25 years between them and substantial differences in background breast cancer incidence, mammographic equipment (film-screen vs digital), retrospective (previous study) vs prospective (current study) design, and laboratory setting (previous study) vs screening practice (current study).

In the current Dutch Breast Cancer Screening Program, the target value for the overall recall rate is < 2.5% [13], which is largely based on recommendations from the study by Otten et al [8]. This recommendation aimed to improve cancer detection by increasing the previous recall rate of 1%. Both the abnormal interpretation rate (2.29%) and the recall rate after double reading (2.32%) observed in our study meet this target value. A further increase of up to 0.5% in the individual recall rate could be considered. However, it must be taken into account that this still adds proportionally more false positives than what is gained in cancer detection. In addition, there is always the risk that radiologists will go too far in lowering their recall threshold and end up with a recall rate that is higher than desired.

The balance between cancer detection and recall rates can be influenced by many different factors, such as the underlying breast cancer incidence, screening interval, quality of screening examinations, mix of initial and subsequent screens, and training of screening radiologists. This means that the results cannot be directly generalized to other settings. For example, breast cancer screening in the US differs greatly from screening in the Netherlands. In two US studies, much higher optimal ranges were identified for the recall rate, namely 12%–14% [14] and 7%-9% [15]. Furthermore, the latter publication was immediately met with a response that spoke of an optimal recall rate of 3%–4% for a specific US center, emphasizing that this is highly setting dependent [16]. A study from the UK, which has a 3-year interval in breast cancer screening, suggested that the optimal recall rate for initial screens was between 4.6% and 7%, whereas for subsequent screens it was between 2.6% and 4% [17]. This is in line with current UK guidelines of less than 7% and less than 3% for initial and subsequent screening examinations, respectively, as the ‘achievable level’ [18]. However, none of these studies derived the optimal recall rates prospectively in actual screening conditions. Therefore, it could be of interest for the ROCS study design to be repeated in other screening programs to gauge their recall–detection trade-offs.

The latest European guidelines (from 2006) that suggest target values have specified recall rates of less than 5% and less than 3% as the ‘desirable standard’ for initial and subsequent screening examinations, respectively [19]. However, these target values are potentially outdated as they were established almost 20 years ago, before the introduction of digital mammography. The newer European Commission Initiative on Breast Cancer guidelines do not provide target values for recall rates [20]. Furthermore, a comparative study of European programs, based on data from 2013 to 2017, showed that there is a lot of variation in screening indicators across Europe [6]. The unadjusted positivity rate, similar to the recall rate, ranged from 2.9% to 19.8% for initial and 1.4% to 9.6% for subsequent screening examinations. Even after correcting for relevant factors, such as age, screening interval, and initial/subsequent ratio, substantial variation remained. According to the authors, differences in background breast cancer risk and screening protocols may have contributed to this variation. This suggests that even in relatively comparable screening programs, such as those within Europe, the balance between these different indicators can be very different. Therefore, it is essential to separately determine the optimal recall rate for each setting. Moreover, if something changes within a screening program, such as the potential future introduction of AI as a detection aid or triage tool, this balance will need to be reassessed as well. Used in this manner, AI has the potential to further improve overall screening performance [21], which may result in a different recall–detection trade-off.

Our study has some limitations. Although the radiologists were instructed to score only the initial first and second readings, it cannot be ruled out that some radiologists still assigned a PoM score during the consensus process. However, given an average discrepancy rate of approximately 2%, this will only involve a few cases, even in the highly unlikely event that all discrepancy readings were included. In addition, we were able to exclude arbitration readings from our dataset. Furthermore, the scores had to be given on a separate tablet instead of in the system where the recall recommendation was entered. With the help of an automatic photograph at the time of entering the PoM score, the correct ID could be linked to the score. However, sometimes, there was still no visible ID, and these cases had to be excluded. Entering the scores directly into the system would have been less labor-intensive in processing the data and less prone to errors. Nevertheless, this workaround was used because of the inability to modify the reading workstation. In addition, no personal data, such as the participant’s age, was disclosed for the screening examinations due to privacy reasons.

As assessed in the ROCS study, Dutch screening radiologists performed with high accuracy (AUC = 0.91) and operated at an appropriate recall-detection trade-off point, suggesting that the current target value within Dutch screening (< 2.5%) does not need to be revised. Ultimately, setting an optimal range for recall rates is a matter of policy that should be informed by an understanding of the trade-off between recall and detection rates within a specific program. A recall rate that is appropriate for the Dutch setting may not be appropriate for other settings. The ROCS study methodology is well-suited as a cost-effective method to evaluate the cancer detection–recall trade-off in various contexts, including beyond the Dutch Breast Cancer Screening Program.

## Supplementary information

Supplementary information

## Abbreviations

AUC: Area under the receiver operating characteristic curve
PoM: Probability-of-malignancy
ROC: Receiver operating characteristic
ROCS: Recall and detection of breast cancer in screening
