# Supplementary material for: Finding the optimal recall rate in breast cancer screening: results from the ROCS study
Source: Eur Radiol. 2026 Mar 5;36(7):5595–603. doi: 10.1007/s00330-026-12370-5 (PMC13282243; doi:10.1007/s00330-026-12370-5)
Supplement: Supplementary file 1 — Supplementary information [file 330_2026_12370_MOESM1_ESM.pdf]

# Finding the optimal recall rate in breast cancer screening: results from the ROCS study

## ELECTRONIC SUPPLEMENTARY MATERIAL

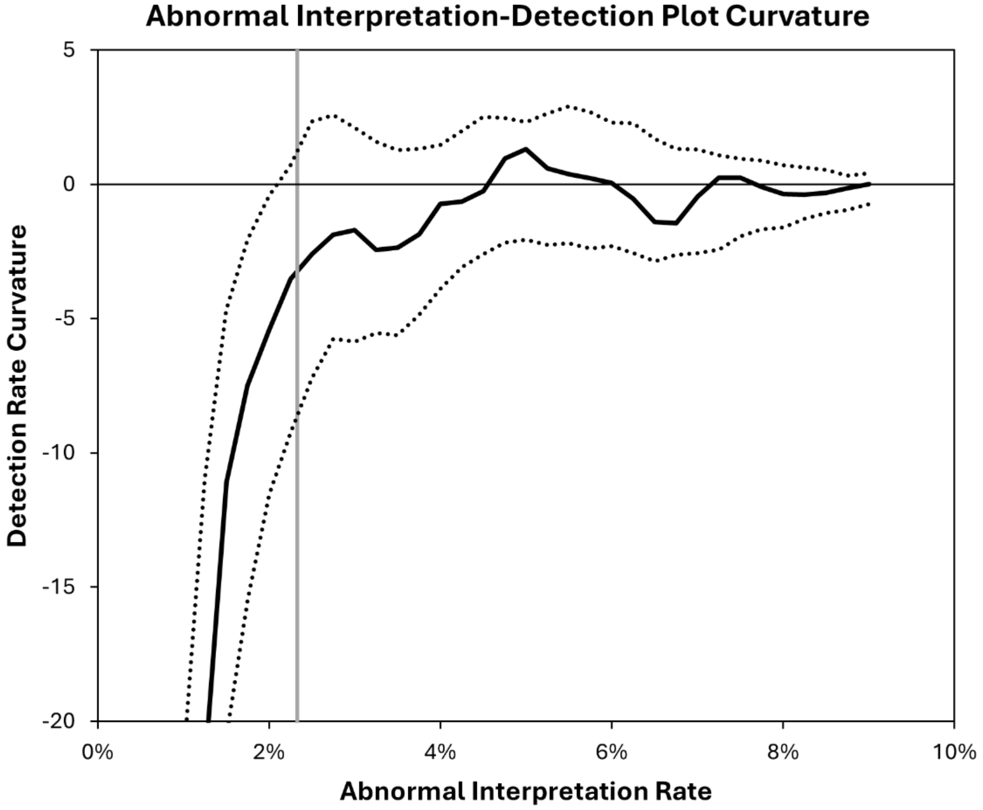


**Supplementary Figure 1**. Curvature of the abnormal interpretation-detection graph (Figure 3A). Curvature (second derivative) indicates how much a function bends at a given point. The average abnormal interpretation rate (dashed vertical line) is located near the end of a region of high negative curvature.

**Methods**

Local curvature of the abnormal interpretation-detection curve was estimated by fitting a quadratic function to data in the neighborhood of a given point (±1% in recall rate). Once the quadratic curve was fit, two times the quadratic coefficient (second derivative of the quadratic function) was used as the estimate of local curvature. This process was repeated across the hierarchical bootstrap sample to obtain a 95% confidence band.

**Description**

This figure shows the curvature of the abnormal interpretation-detection graph (Figure 3A). Curvature (second derivative) indicates how much a function bends at a given point. Positive curvature means the abnormal interpretation-detection curve is bending upward, while negative curvature means it is bending downward. This figure shows that the operating point of the radiologists (dashed vertical line) is located right at the end of the region of high negative curvature. In other words, compared to the current operating point of the radiologists, if the abnormal interpretation rate (i.e., the individual recall rate) decreases, the detection begins to decrease rapidly. On the other hand, moving the operating point to the right, i.e., increasing the abnormal interpretation rate, results in little change in the detection rate. This suggests that readers are at their lowest recall level before they start to substantially lose sensitivity.
